# From the European Youth Olympics Festival to professional sport. The level of athlete dropout – a longitudinal cohort study

**DOI:** 10.3389/fpsyg.2025.1572597

**Published:** 2025-05-02

**Authors:** Krisztina Kovács, Dorottya Pignitzky, Csaba Bartha, Johanna Takács

**Affiliations:** ^1^Department of Psychology and Sport Psychology, Institute of Economic and Social Sciences, Hungarian University of Sports Science, Budapest, Hungary; ^2^Hungarian Olympic Committee, Budapest, Hungary; ^3^Institute of Sport, Hungarian University of Sports Science, Budapest, Hungary; ^4^Department of Social Sciences, Faculty of Health Sciences, Semmelweis University, Budapest, Hungary

**Keywords:** dropout, youth sport, EYOF, RAE, junior-to-senior transition

## Abstract

**Background:**

Globally, the dropout rate among young athletes, which is influenced by a range of complex and interconnected factors, tends to rise significantly when adolescence starts.

**Objective:**

The present retrospective cohort study aimed to explore the characteristics and extent of dropout in youth sports in Hungary.

**Methods:**

We analysed the status of 409 athletes who participated in the summer editions of the European Youth Olympic Festival (EYOF) between 2009 and 2019, assessing their athletic status 5 years later. Specifically, we examined the associations between dropout rates and factors such as gender, types of sport and relative age quartiles.

**Results:**

Our findings reveal that dropout rates were higher in individual sports compared to team sports. Additionally, within individual sports, athletes born in the second age quartile had a slightly higher dropout rate, while those born in the third quartile experienced a slightly lower rate compared to the other quartiles.

**Conclusion:**

These insights contribute to a deeper understanding of the factors influencing dropout in youth sports.

## 1 Introduction

One of the primary aims of youth sport is to foster a committed generation of athletes and create a successful pool of professional athletes. Athlete development models such as the Developmental Model of Sport Participation (Côté and Hay, 2002) and the Holistic Athletic Career Model (Wylleman and Lavallee, 2004) emphasize the importance of fostering positive youth development, which can enhance long-term commitment and reduce the likelihood of dropout. Within temporary cessation of sports participation, researchers distinguish between “dropoff” and dropout (Côté, 1999), the former involving a one-year break, after which athletes return to regular physical exercise, while the latter means that an athlete does not register for two consecutive years or competition seasons (Moulds et al., 2022).

### 1.1 Factors associated with dropout in youth sport

The underlying mechanisms behind dropout from organized sports are multifactorial. Bronfenbrenner’s Bioecological Theory of Human Development (Bronfenbrenner, 1995; Bronfenbrenner and Morris, 1998; Moulds et al., 2022) provides a useful theoretical framework for understanding athletic dropout. According to this model, dropout can be influenced not only by an athlete’s bio-psycho-social characteristics (e.g., gender, age, physical abilities, motivation) but also by interactions with their immediate (microsystem: family) and broader environments (mesosystem: sports school; exosystem: club, associations; macrosystem: sports policy strategies) (Carlman et al., 2013; Sabo and Veliz, 2008; Witt and Dangi, 2018). Different factors may characterise dropout in individual sport versus team sports (Back et al., 2022a; Baron-Thiene and Alfermann, 2015), while gender differences are also evident, with higher dropout rates among females than males (Lattore-Román et al., 2018; Murray and Sabiston, 2022). A longitudinal cohort study among adolescent girls showed that enjoyment mediated the relationship between social identity and sport dropout (Murray and Sabiston, 2022).

Witt and Dangi (2018) distinguished three types of constraints that may lead to youth sports dropout: intrapersonal (e.g., the lack of enjoyment, low perceived competence, stress), interpersonal (e.g., parental pressure, the lack of time for other activities), and structural (e.g., injuries, burnout, cost). Fraser-Thomas et al. (2008) highlighted the roles of significant others (e.g., coaches, parents, peers, siblings) in influencing dropout, identifying factors such as limited one-on-one coaching, pressuring parents, and the lack of peers and sibling rivalries. Boiché and Sarrazin (2009) found that social factors and psychological attributes such as the perceived value of the activity and parental investment were predictors of dropout among French adolescent athletes. Similarly, a qualitative study among Czech and Portuguese athletes came to the conclusion that the loss of enjoyment, negative team atmosphere, financial difficulties and conflict between sport and school were significant contributors to dropout (Silva Dias et al., 2018).

Enoksen (2011) emphasized factors such as injuries, school priorities and the lack of motivation as leading causes of dropout among track and field athletes. Temple and Crane (2015) expanded on the contextual factors associated with dropout, including the impact of the relative age effect (RAE). RAE describes a biased distribution of elite athletes’ birthdates, with more athletes having been born at the beginning of a competitive year (e.g., in September) or a biannual competition cycle and fewer athletes at the end (e.g., in August). Similarly, Sandercock et al. (2014) highlighted the importance of RAE in the selection and cessation of sport, finding that children born in autumn were fitter, stronger and more powerful than those born in summer. Dugdale et al. (2021) confirmed the presence of RAE among youth football players, even though studies suggest that RAE diminishes as players age and transition to professional levels (González-Víllora et al., 2015; Dugdale et al., 2021). RAE has been identified as a significant factor influencing athlete dropout. Research examining French swimmers revealed that birth quarter-based dropout differences peaked at age 13 among females (10%) and age 15 among males (8.1%) (Difernand et al., 2025). Likewise, an analysis by Moulds et al. (2022) demonstrated that the relative age effect reaches its peak in athletes between 13–16 years. Nevertheless, the influence of RAE might differ across sports. Côté et al. (2006) for example found that it existed in hockey and baseball but not in basketball or golf.

### 1.2 Dropout rates in youth sport

Several international studies have examined the rates of athletic dropout, concluding that the dropout rate among adolescent athletes is significantly higher than at any other age. In a study on football players aged 13 to 17 years, Calvo et al. (2010) found that the lack of self-determined motivation was associated with higher dropout rates. An Australian study shows that the participation rates peak at 45–46% for athletes aged 5–14, before dropping to merely 23% in the 15–19 age group (Eime and Harvey, 2018). Vella et al. (2020) found that in Australia, approximately one-third of youths drop out of sports between the ages of 10 and 14, and in America, the dropout rate is estimated at 45% among high school students (Sabo and Veliz, 2008). In a systematic review, Møllerløkken et al. (2015) reported that the annual dropout rates in football remain stable between the ages of 10 and 19, with a higher rate for girls (26.8%) compared to boys (21.4%). These dropout rates show similarities internationally, both in proportions and in characteristics (Carlman et al., 2013; Côté et al., 2006; Vella et al., 2020), with surveys highlighting the vulnerable situation of girls, urban youths (birthplace bias towards smaller cities - professional athletes are more likely to come from smaller cities with a population under 500,000) and children from lower-income families. While age has been identified as one of the most significant risk factors for dropout, the reasons for dropout are multifaceted. Specifically, participation in organized sport tends to be more inclusive for boys than girls (Back et al., 2022b; Espedalen and Seippel, 2024). Female athletes often face gender stereotypes, heightened weight-related pressure, body dissatisfaction and disordered eating (Crane and Temple, 2015). They also experience more negative coaching practices and microaggression compared to their male counterparts (Roberts and Quesnel, 2023). These factors may contribute to increased dropout rates among female athletes. Additionally, parental sociodemographic factors such as a lower level of education, financial constraints and a migrant background are linked to higher youth sport dropout rates (Moulds et al., 2022). Many young athletes from less resourceful families may initially engage in sports but struggle to continue as costs for tournaments, travel, and equipment increase (Espedalen and Seippel, 2024). Dropout also significantly impacts talented youth athletes. A study on youth elite football academies and national teams in Germany found that the likelihood of no longer being part of talent identification (TID) and long-term nurture in talent promotion programmes was over 50% after 3 years and over 70% after 5 years (Güllich, 2013).

Unfortunately, specific data on dropout in Hungary are lacking. A survey on the competitive sports strategy of Olympic sports in Hungary (Hungarian Olympic Committee, 2022a) shows that the dropout rate is excessively high. A high dropout rate can be critical, particularly in the field of youth sport, and one of the most significant youth sport event is the European Youth Olympic Festival (the EYOF). The EYOF is a bi-annual multi-sport event for European athletes aged 14 to 18, having been organized since 1991. The core program includes 9 to 10 sports and the host country has the option to add additional sports. The European Olympic Committees, in collaboration with continental and international federations, oversee the event’s organization, including decisions on the competition format, events, and age categories. Each sport at the EYOF is governed by its own distinct set of regulations, qualification criteria and age requirements for participant eligibility. The summer edition of the EYOF is always held at the end of July. Its primary objective is to introduce young athletes to the Olympic framework, providing a foundational experience that promotes their development and progress in elite sports. Participation is open to all European athletes nominated by their respective National Olympic Committees. National selection processes are carried out by the national federations. The selection is mostly based on the national ranking position and the results of youth athletes.

The present study aimed to measure the dropout rate among youth athletes who participated in the EYOF between 2009 and 2019, focusing on identifying demographic (gender, age) and sport-related (types of sport, current competitive status) factors.

## 2 Methods

### 2.1 Procedure

The current retrospective cohort research is part of a larger study, in which the characteristics of dropout from sport in Hungary are investigated. Information on the athletes, including their birth dates, competitive category, national team membership and when they ceased to compete professionally, is generally accessible to the public (Hungarian Olympic Committee, 2023b). The database was provided and anonymized by the Hungarian Olympic Committee and contained information about athletes who participated in the summer games of the European Youth Olympics Festival (EYOF). The dataset included the athletes’ gender, date of birth, types of sport, year of the EYOF participation and their status 5 years later. The data were collected between 2009 and 2019. The research was conducted in accordance with the Declaration of Helsinki and was approved by the Research Ethics Committee of the Hungarian University of Sport Science, with license number TE-KEB/06/2024.

### 2.2 Participants

409 Hungarian athletes participated in the EYOF from 2009 to 2019. The youth athletes (172 males and 230 females) were between the ages of 14 and 18. Relative age quartiles were determined by creating four quartiles (Q) based on the athletes’ birth month: Q1 = 1st January to 31st March; Q2 = 1st April to 30th June; Q3 = 1st July to 30th September; and Q4 = 1st October to 31st December. In terms of the birth quartiles of age, 32.6% of the athletes were born in Q1, 24.9% in Q2, 24.6% in Q3 and 17.9% in Q4.

The athletes engaged both in team sports (*n* = 150; basketball = 14.4%, volleyball = 6%, handball = 16.9%) and in individual sports (*n* = 252; athletics/track and field = 25.6%, judo = 13.4%, artistic gymnastics = 8.5%, swimming = 4%, cycling = 4%, tennis = 3.5%, wrestling = 2.2% and canoeing, kayaking = 1.5%). Broken down to the years of the EYOF, 12.2% of the athletes participated in 2009, 6.2% in 2011, 14.4% in 2013, 15.9% in 2015, 32.8% in 2017 (hosted by Hungary) and 18.4% in 2019. Sports dropout is defined as the cessation of participation in competitive sports. Consequently, athletes who neither engaged in professional sports nor registered in organized sports for 5 years following the EYOF were classified as dropped out. After 5 years of the EYOF, 35.5% of the athletes have dropped out from competitive sport (not reaching professional sport level).

### 2.3 Statistical analysis

The data analysis included a frequency analysis of gender (male/female), the types of sport (individual/team), relative age quartiles (Q1-Q4) and athlete status 5 years after the EYOF (having a professional athlete career or having dropped out from competitive sport). To examine the associations between the athletes’ status (professional athlete career vs. having dropped out from competitive sport) and the types of sport (individual vs. team) crosstabs were used with Pearson chi-square tests or with Fisher’s exact test. The effect of gender and RAE were examined using stratification. Cramer’s V or Phi measurements were calculated to estimate the strength of the associations. The level of significance was set at 0.05. All statistical analyses were performed with IBM SPSS Statistics for Windows, Version 26.0 (IBM Corp. Released 2019. Armonk, NY).

## 3 Results

Dropout and the types of sport showed a significant, strong association [*χ*^2^ (1, *N* = 402) = 51.636, *p* < 0.001, V = 0.36]. A lower proportion of team sport athletes dropped out (*n* = 20, 13.3%) compared to individual athletes (*n* = 123, 48.8%).

**Figure 1:**
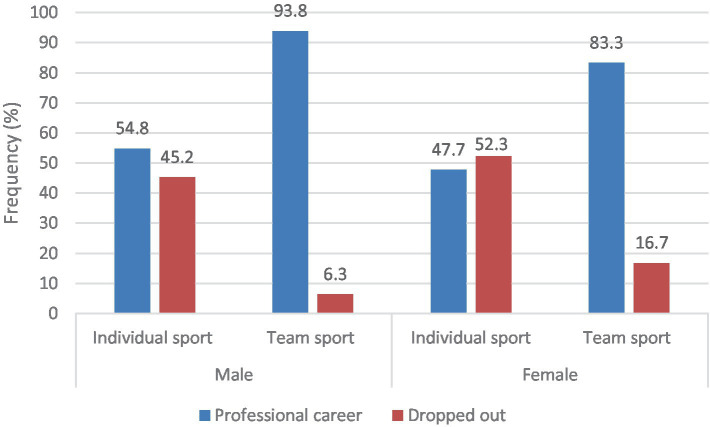
The association between athlete status and the types of sport by gender.

A significant, strong association was found both in males [χ^2^ (1, *N* = 172) = 23.249, *p* < 0.001, φ = −0.37] and females [*χ*^2^(1, *N* = 230) = 31.166, *p* < 0.001, φ = −0.37]. Regardless of gender, a lower proportion of team athletes dropped out compared to individual athletes (Figure 1).

**Figure 2:**
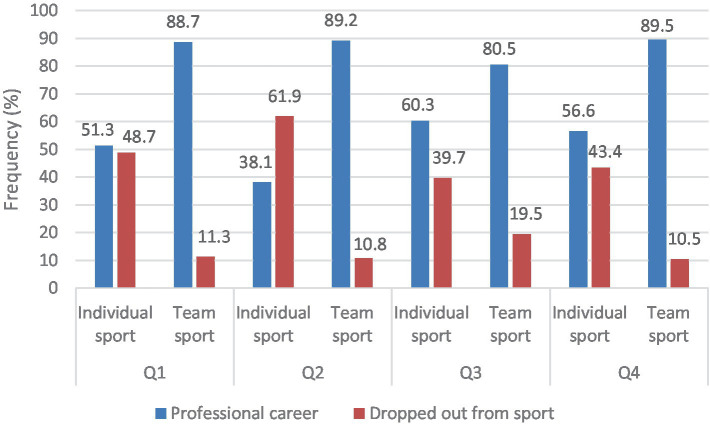
The association between athlete status and types of sport by RAE. Q1 = January, February, March; Q2 = April, May, June; Q3 = July, August, September; Q4 = October, November, December.

Our results showed a significant association between athlete status and the types of sport in all age categories [*χ*^2^ (1, *N* = 402) = 51.636, *p* < 0.001, Φ = 0.36]. A higher proportion of individual athletes drop out compared to team athletes. Based on the strength of association and the frequency data, the Q2 age group showed the highest proportion of dropout among individual athletes [*χ*^2^ (1, *N* = 100) = 24.828, *p* < 0.001, Φ = −0.50, *n* = 39]. In the Q1 [*χ*^2^ (1, *N* = 131) = 19.786, *p* < 0.001, Φ = −0.39, *n* = 38] and Q4 [*χ*^2^ (1, *N* = 72) = 6.667, *p* = 0.011, Φ = −0.30, *n* = 23] age groups, nearly half of the individual athletes dropped out, which is a higher proportion compared to team athletes. The lowest dropout rate among individual athletes was found in the Q3 age group [*χ*^2^ (1, *N* = 99) = 4.531, *p* = 0.047, Φ = −0.21, *n* = 23] (Figure 2).

## 4 Discussion

The purpose of this retrospective cohort study was to determine the dropout rate for athletes who were selected for EYOF. Furthermore, the study also aimed to examine the associations between gender, types of sport, RAE and sport dropout in youth sport. The inclusion criterion was participation in the EYOF between 2009 and 2019. The participants selected for this study were qualified, i.e., they can be considered the most skilled and successful athletes in their age group (Hungarian Olympic Committee, 2022b, 2023a).

Based on the self-determination theory, the basic need for competence can be described as a “feeling of mastery, a sense that one can succeed and grow” (Ryan and Deci, 2020, p. 1.), which can be reflected in one’s results or in being selected into the national team. Some studies highlight the importance of self-determined motivation, which is associated with a lower level of dropout (Back et al., 2022a; Baron-Thiene and Alfermann, 2015; Calvo et al., 2010). On the other hand, other studies point out that early talent identification and success do not prevent young people from dropping out (Fraser-Thomas et al., 2008; Güllich, 2013). The junior-to-senior transition (JST) plays the most critical role in athletes’ overall career, which is especially challenging for talented juniors who achieve early success and recognition (Stambulova et al., 2009, 2017, 2021). At the same time, researchers emphasize the fact that early performance expectations and pressure from the youth athletes’ sport environment are associated with burnout and dropout (Barreiros et al., 2014; Gould et al., 1996). Although in our research the participants were successful as youth athletes, 35.5% of them did not reach a professional sport level and dropped out, which is similar to the dropout trends in other countries (Carlman et al., 2013; Vella et al., 2020).

Our findings suggest that individual athletes exhibit a higher attrition rate in comparison to team athletes. It is noteworthy that in Hungary, while team sports offer various opportunities for financial support (initially through scholarships and later through salaries) for junior athletes, such options are considerably more limited for individual athletes. The Hungarian government provides financial support to team sports through a type of corporate tax (TAO), which increases athletes’ opportunities for training, and their access to equipment, and provides teams with the opportunity to launch another adult team besides the first league, where youth athletes can participate (Act LXXXI of 1996 on Corporate Tax and Dividend Tax (TAO), n.d.; Government Decree, n.d.). Based on the sport-commitment model (Scanlan et al., 1993, 2016), enthusiastic commitment requires valuable opportunities and a fair proportion of personal investment. As a result of the higher amount of tangible support, youth athletes involved in team sport are given better opportunities (e.g., job opportunities, competitions and training camps abroad, medical staff and sport psychologists). By contrast, individual athletes have to invest more in their sport and their career, which may weaken their commitment. Even though individual sports can be associated with a higher level of dropout, and tangible support may determine the level of athletes’ commitment, it is important that our results are applied in practice in a constructive way. The funding invested in youth sports should not focus on immediate success but should contribute to long-term development instead.

Contrary to previous studies (Crane and Temple, 2015; Difernand et al., 2025; Espedalen and Seippel, 2024; Lattore-Román et al., 2018), this research found no significant differences in dropout rates between female and male athletes. Existing literature highlights that factors contributing to higher dropout rates among females are often linked to gender stereotypes and roles (Chalabaev et al., 2013; Crane and Temple, 2015; Persson et al., 2020). In this study, female participants demonstrated success in their respective sports, qualified for the EYOF, and were members of the national junior team. It is assumed that as sports become more competitive, gender disparities may diminish, and accessibility in sports becomes more performance-oriented rather than gender-based. Further investigation is needed to examine gender-specific constraints influencing dropout in organized sport.

In line with the results of a study by Sandercock et al. (2014), we found a significant association between dropout rates and relative age quartiles: individual athletes born in Q2 had a higher dropout rate in comparison to other quartiles. In Hungary, the transition between age categories is mostly based on academic years. There is a practice that offers talented youth athletes the opportunity to train and participate in competitions in older age categories at the beginning of the new school year (in September). This practice highly depends on the rules and regulations of the specific sport and is more prevalent in team sports, which may have implications for our results. For example, in the U16 basketball championships, a maximum of four U14 players are permitted to participate per match (Hungarian Basketball Federation, 2024). While in handball, a U16 player is allowed to hold up to three competition licenses at the same time, based on different rules compared to basketball. These licences may include two different youth age categories and one adult age category (Hungarian Handball Federation, 2024). While competing in older age categories is more regulated in individual sports (Hungarian Athletics Association, 2024), in team sports, this policy is more flexible. Taking into consideration previous research results, it is important to highlight that RAE might diminish with age and when athletes reach the professional level (González-Víllora et al., 2015; Dugdale et al., 2021). Further research is needed to determine why drop-out rates based on RAE are lower in team sports compared to individual sports.

### 4.1 Limitations and future research

As our study is a retrospective cohort database analysis, it is crucial to emphasize the limitations of this study concerning the complexity and multifactorial characteristics of dropout. We would like to highlight the fact that the EYOF has a limited number of sports compared to the Olympic games, which may have implications for our results. It is important to point out that the EYOF can be the first international competition experience for many athletes, and this may have an effect on the athletes’ sport career and commitment. To reinforce our results, a longitudinal study is necessary to monitor cessation from sport over time. Our study did not address measuring the commitment and motivational style of athletes. Further investigation is required to establish the connection between the dropout level of talented and successful athletes and tangible support. The factors leading to dropping out from sports are diverse and multifaceted. It is important to note that this study did not aim to examine the different causes of dropout or focus on individual differences.

### 4.2 Implications

This study focuses on the characteristics of dropout and the successful transition into professional sport among athletes who participated in the EYOF. Our results show that both the types of sport and relative age quartiles are associated with dropout. Individual athletes and athletes born in Q2 tend to have a higher dropout rate compared to team athletes or athletes born in other quartiles. Our findings suggest that this phenomenon may be based on the characteristics of the Hungarian sport support system, which requires further investigation. Such a level of dropout in individual sport receiving priority state support suggests that there are professional and organizational problems in the background, such as the lack of coaches. These findings help to understand the complexity of the phenomenon of dropout in youth sport and have important implications for the training of sport professionals such as coaches, sports medicine physicians and rehabilitation experts.

## Data Availability

The raw data supporting the conclusions of this article will be made available by the authors, without undue reservation.
